# Contribution of IQ in young adulthood to the associations of education and occupation with cognitive ability in older age

**DOI:** 10.1186/s12877-021-02290-y

**Published:** 2021-06-05

**Authors:** Insa Feinkohl, Petra Kozma, Friedrich Borchers, Simone J. T. van Montfort, Jochen Kruppa, Georg Winterer, Claudia Spies, Tobias Pischon

**Affiliations:** 1 Max-Delbrueck-Center for Molecular Medicine in the Helmholtz Association (MDC), Molecular Epidemiology Research Group, Robert-Rössle Str. 10, 13092 Berlin, Germany; 2 Charité - Universitätsmedizin Berlin, corporate member of Freie Universität Berlin and Humboldt-Universität zu Berlin, Berlin, Germany; 3 University Medical Center Utrecht Brain Center, Utrecht, the Netherlands; 4 Berlin Institute of Health (BIH) at Charité - Universitätsmedizin Berlin, Berlin, Germany; 5 Institute of Medical Informatics, Charité - Universitätsmedizin Berlin, Corporate Member of Freie Universität Berlin and Humboldt-Universität zu Berlin, Berlin, Germany; 6 Max-Delbrueck-Center for Molecular Medicine in the Helmholtz Association (MDC), Biobank Technology Platform, Berlin, Germany; 7 Berlin Institute of Health (BIH) at Charité - Universitätsmedizin Berlin, Core Facility Biobank, Berlin, Germany

**Keywords:** Cognitive epidemiology, Cognitive ageing, Education, Occupation, Pre-morbid IQ

## Abstract

**Background:**

Studies suggest that a higher education and occupation are each associated with a higher late-life cognitive ability, but their inter-relationships in their association with cognitive ability and the contribution of peak IQ in young adulthood (‘pre-morbid IQ’) often remain unclear.

**Methods:**

Cross-sectional analysis of 623 participants aged ≥65 years of the BioCog study. Education was coded according to the International Standard Classification of Education (ISCED; range 1 to 6). Occupation was coded as ‘semi/unskilled’, ‘skilled manual’, ‘skilled non-manual’, ‘managerial’, ‘professional’. A summary score of global ability (‘*g*’) was constructed from six cognitive tests. Pre-morbid IQ was estimated from vocabulary. The Geriatric Depression Scale assessed symptoms of depression. Age- and sex-adjusted analyses of covariance were performed.

**Results:**

Education (partial eta^2^ 0.076; *p* < 0.001) and occupation (partial eta^2^ = 0.037; p < 0.001) were each significantly associated with *g*. For education, the association was attenuated but remained statistically significant when pre-morbid IQ was controlled for (partial eta^2^ 0.036; *p* < 0.001) and was unchanged with additional adjustment for depression (partial eta^2^ 0.037; *p* < 0.001). For occupation, the association with *g* was no longer significant when pre-morbid IQ (partial eta^2^ = 0.015; *p* = 0.06) and depression (partial eta^2^ = 0.011; *p* = 0.18) were entered as covariates in separate steps. When education and occupation were entered concurrently into the fully adjusted model, only education was independently associated with *g* (partial eta^2^ 0.030; *p* < 0.001; occupation, *p* = 0.93).

**Conclusion:**

While a higher education and a higher occupation were each associated with a higher late-life cognitive ability, only for education some unique contribution to cognitive ability remained over and above its relationship with pre-morbid IQ, depression, and occupation. Further research is needed to address whether a longer time spent in education may promote late-life cognitive ability.

**Supplementary Information:**

The online version contains supplementary material available at 10.1186/s12877-021-02290-y.

## Background

Neuropathological hallmarks of age-related cognitive impairment, such as an accumulation of beta amyloid plaques in the brain or cerebrovascular degeneration, are well-described, but their presence does not always correlate with clinical manifestation. Given the same degree of neuropathological damage, some people show debilitating symptoms whereas others do not [1]. The ‘cognitive reserve’ concept aims to resolve this discrepancy and suggests inter-individual differences in the ability to buffer the functional and behavioral consequences of cerebral neuropathology.

Cognitive reserve is commonly quantified by the peak level of cognitive ability in young adulthood prior to the onset of any age-related cognitive decline or dysfunction, termed ‘pre-morbid IQ’. Studies with data on pre-morbid IQ measured in young adulthood are rare, but pre-morbid IQ can additionally be approximated by vocabulary at any point throughout the lifespan, because the vocabulary domain is immune to age-related decline [2–4] and remains intact even in mild to moderate dementia [5]. Sociobehavioral measures such as self-reported educational and occupational attainment [6], income [7, 8], car ownership [8] or childhood home occupancy rate [9] or composites thereof [10] can also serve as proxies for cognitive reserve, so that the research field is characterized by a substantial heterogeneity [11]. Although all of these parameters are subject to an influence by societal and cultural constraints, historical context, and opportunity, introducing a noise into their statistical analysis, associations with risk of dementia [12–15] and milder forms of impairment [9, 16–18] are frequently found. Analyses additionally addressing several cognitive reserve parameters within the same study, which allow researchers to determine any inter-relationships among them in their associations with late-life cognition, are rarer, however. This is a relevant oversight, because unless for instance pre-morbid IQ is considered as a potential confounder in analyses of education or occupation and cognition in older age, we cannot draw conclusions as to any potential protective effects of these modifiable factors. In fact, some previous studies have reported that associations of a higher education with reduced odds of cognitive impairment or reduced rate of cognitive decline were rendered statistically non-significant or became less consistent once pre-morbid IQ was controlled for [19–21], which warrants further investigation.

Here, we used a sample of older adults scheduled for surgery to determine the associations of self-reported educational and occupational attainment with their level of cognitive ability. We additionally assessed the contribution of pre-morbid IQ estimated from vocabulary to the hypothesized associations through statistical adjustment.

## Method

### Study design

Baseline data from the Biomarker Development for Postoperative Cognitive Impairment in the Elderly (BioCog) study [22] were used for a cross-sectional analysis. Briefly, 1033 patients scheduled for surgery were enrolled at three hospital sites in Berlin, Germany, and Utrecht, the Netherlands between 2014 and 2017. Inclusion criteria were, among others, age ≥65 years, Mini Mental State Examination (MMSE) ≥24 [23], and no history of neuropsychiatric disease or addiction disorder. We only included native speakers of German and Dutch in the present analysis. All participants gave written informed consent to participate in the study.

### Education and occupation

Full educational background including any degrees attained was self-reported during interview and classified according to the International Standard Classification of Education (ISCED). At the Dutch study center, the ISCED 2011 classification system was used [24]; at Berlin, ISCED 1997 was used [25]. Results for the Dutch center were later converted to ISCED 1997 based on the ISCED 1997–2011 conversion table [24]. ISCED 1997 assigns individuals categories ranging from 0 to 6 (0, ‘pre-primary’; 1, ‘primary’; 2, ‘lower secondary’; 3, ‘upper secondary’; 4, ‘post-secondary non-tertiary’; 5, ‘first stage tertiary’; 6, ‘second stage tertiary’). For the purpose of the present analysis, participants were grouped into ‘ISCED 1/2’, ‘ISCED 3/4’, and ‘ISCED 5/6’ (no participant had ISCED 0). Results reported in this manuscript were similar when ISCED was used as the original six-level variable (range 1 to 6; data not shown). Last occupation before retirement, or (if not retired) current occupation, was self-reported and later categorized for all study centers by a single researcher (PK) according to the following groups: ‘semiskilled/unskilled’, ‘skilled manual’, ‘skilled non-manual’, ‘managerial’ and ‘professional’ [26].

### Pre-morbid IQ

Pre-morbid IQ was estimated from vocabulary [2–5]. Differences between the German and the Dutch language necessitated use of two different tests for Berlin and Utrecht study sites. Participants at Berlin performed the 'Mehrfachwortschatztest A' (MWT-A), the German version of the Mill-Hill Vocabulary Scale (MHVS) [27, 28]. They were presented with 37 items, ordered in ascending level of difficulty. Eeach item consisted of a set of 5 words of which 4 were distractors and 1 was the target word. For each item, participants were instructed to identify the one word among the set of words that they knew. One point was given for each correctly identified word. There was no time constraint; thus participants completed the test at their own pace. At Utrecht, the Dutch Adult Reading Test (DART) [29] was used. Participants read out a list of 100 irregularly pronounced words to a study physician or nurse. One point was given for each correctly pronounced word. Total scores for the MWT-A and DART were each constructed by summing up the points achieved for each item; thus, possible MWT-A scores ranged from 0 to 37, and possible DART scores from 0 to 100. Both tests have been found to be free from ceiling effects [5, 30], including in the present study (MWT-A score range 11 to 37 with 1 individual obtaining the full score; DART score range 32 to 100 with 4 individuals obtaining the full score in the present analysis samnple) MWT-A and DART scores were then converted to intelligence quotient (IQ) ranks based on published norms [29, 31]. IQ is constructed based on performance of representative samples and has a mean of 100 with standard deviation (SD) of 15. An MWT-A score of 27 and a DART score of 70 each correspond to an IQ of 100 for instance. Following this conversion, IQ scores derived from MWT-A and DART were merged and used as a single variable (‘pre-morbid IQ’; score range 69 to 143 in the present sample).

### Level of cognitive ability

Participants completed six cognitive tests that tapped several age-sensitive domains including processing speed, executive function and memory as detailed previously [32]. Four were computerized and performed on hand-held devices (Paired Associates Learning; Spatial Span; Simple Reaction Time; Verbal Recognition Memory from the CANTAB® battery, Cambridge Cognition Ltd.); two were speeded conventional tests (Grooved Pegboard; Trail-Making Test-B). People who perform well on one cognitive test are likely to also perform well on other tests [33], because performance reflects a single latent underlying global ability factor termed ‘*g*’ [34]. The factor *g* has the advantage that it accounts for any measurement error that inevitably affects each individual cognitive test [35]. We thus calculated *g* for the full BioCog sample from the six age-sensitive cognitive tests using principal component analysis (PCA) with extraction of factors with Eigenvalue >1. The factor *g* has a mean of 0 with standard deviation of 1 and explained 39.5% of variance in scores on the six cognitive tests [36].

### Mood examination

The 15-item Geriatric Depression Scale (GDS) screened for symptoms of depression such as a loss of interest in previously enjoyed activities or lack of energy (score range 0 to 15; high scores mean more depressive symptoms; sample item ‘do you feel that your life is empty?').

### Statistical analysis

Depression scores were natural log-transformed due to a skewed distribution. Analyses of variance (ANOVA) were used to compare age, pre-morbid IQ and depression scores across education and occupation groups. Chi^2^ test examined the associations of education with occupation, and their associations with sex. For the main analyses, analyses of covariance (ANCOVA) were used to compare mean *g* according to education and occupation categories, respectively. For each, Model 1 controlled for age and sex. Sex was selected as a covariate, because it may influence cognitive function and ageing [37–39] as well as educational [40] and occupational attainment [41, 42] owing to social constraints. Model 2 additionally controlled for pre-morbid IQ. Model 3 additionally controlled for depression scores, as depression is tightly interlinked with cognitive impairment in older age [43] and may also be associated with education and occupation [44, 45]. A final model saw concurrent inclusion of both education and occupation to determine the relative independence of each in their associations with *g*. For each model, we calculated partial eta^2^ to estimate the variance explained by the respective independent variable [46]. Each model was followed by pairwise comparison of mean *g* among education and occupation groups, respectively. The level of statistical significance was set at *p* < 0.05 in these analyses.

Model 3 was subsequently repeated six times with the six individual cognitive tests as dependent variables; the results from these analyses are shown as Supplemental Data. To account for multiple testing, the level of significance was set at a Bonferroni corrected *p* < 0.004 in these analyses. Analyses were performed using SPSS (version 22).

## Results

### Sample characteristics and covariate structure

Six hundred twenty-three participants (aged 65 to 90 years) had complete data on education, occupation and *g*, and provided the analysis sample. Demographic and cognitive characteristics of the full sample and according to educational level are summarized in Table 1. Participants were of a relatively high education, with 42.9% scoring in the highest education group (ISCED 5/6), and had a relatively high pre-morbid IQ (mean 112). Depression scores were relatively low (median GDS 1; interquartile range 0 to 3). 77 (13.2%) of 581 participants with GDS data had mild depressive symptoms (GDS 5 to 9) and 7 (1.2%) had moderate/severe depressive symptoms (GDS > 9) [47]. Females had higher depression scores compared with males (*p* = 0.002), whereas pre-morbid IQ (*p* = 0.74) and *g* (*p* = 0.87) were similar in males and females.

**Table 1:** Sample characteristics in total sample and according to education groups

	Total sample	ISCED 1/2	ISCED 3/4	ISCED 5/6
*N* (% of total sample)	623	94 (15.1%)	262 (42.1%)	267 (42.9%)
Age (years), mean ± SD	72.1 ± 4.9	72.5 ± 5.3	72.4 ± 4.7	71.7 ± 4.9
Male, *n* (%)	363 (58.3%)	49 (52.1%)	124 (47.3%)	190 (71.2%)
Geriatric Depression Scale*, median (interquartile range)	1 (0–3)	1 (1–2)	1 (0–3)	1 (0–2)
Occupation				
Semi−/unskilled, *n* (%)	57 (9.1%)	21 (22.3%)	27 (10.3%)	9 (3.4%)
Skilled manual, *n* (%)	114 (18.3%)	28 (29.8%)	68 (26.0%)	18 (6.7%)
Skilled non-manual, *n* (%)	224 (36.0%)	35 (37.2%)	125 (47.7%)	64 (24.0%)
Managerial, *n* (%)	61 (9.8%)	8 (8.5%)	20 (7.6%)	33 (12.4%)
Professional, *n* (%)	167 (26.8%)	2 (2.1%)	22 (8.4%)	143 (53.6%)
Pre-morbid IQ, mean ± SD	112.0 ± 14.3	101.6 ± 14.1	109.3 ± 13.6	118.4 ± 11.9
G**, mean ± SD	0.06 ± 1.00	− 0.27 ± 1.03	− 0.14 ± 0.98	0.38 ± 0.93

*ISCED* International Standard Classification of Education, *SD* standard deviation. *n = 581 with data. **g was standardized on the full BioCog cohort

Education was not statistically significantly associated with age (partial variance explained, 0.6%; *p* = 0.17; all pairwise comparisons *p* > 0.05) but was statistically significantly associated with sex (*p* < 0.001). For instance, 52.3% of males but 29.6% of females were in the group with the highest level of education (ISCED 5/6). Education was also significantly associated with pre-morbid IQ (partial variance explained, 18.0%; p < 0.001; all pairwise comparisons *p* < 0.001) and with depression scores (partial variance explained, 1.2%; *p* = 0.03). Participants in ISCED 3/4 had higher depression scores (geometric mean 1.42) compared with participants in ISCED 5/6 (geometric mean 1.08; *p* = 0.01; all other pairwise *p* > 0.05; ISCED 1/2 geometric mean 1.33).

Occupation was not significantly associated with age (partial variance explained by occupation, 0.2%; *p* = 0.84; all pairwise comparisons *p* > 0.05) but varied significantly according to sex (*p* < 0.001). For instance, 32.5% of males and 18.8% of females were professionals. Occupation was also statistically significantly associated with pre-morbid IQ (partial variance explained, 13.0%; *p* < 0.001; all pairwise comparisons *p* < 0.001) and with depression scores. Semi/unskilled workers had higher depression scores (geometric mean 1.61) compared with managerial workers (geometric mean 0.70; *p* < 0.001) and professionals (geometric mean 1.08; *p* = 0.03). Participants in the skilled manual group had higher depression scores (geometric mean 1.46) relative to managerial workers (*p* = 0.001), and skilled nonmanual workers had higher depression scores (geometric mean 1.41) compared with the managerial group (*p* < 0.001). The latter had lower depression scores than professionals (geometric mean 1.08; *p* = 0.04; all other pairwise *p* > 0.05). Education and occupation were significantly associated with one another (*p* < 0.001; see Table 1).

### Association of education with cognitive ability

Education was statistically significantly associated with *g* and accounted for 7.6% of its variance in a model controlling for age and sex (Model 1; Table 2). In pairwise comparison, *g* was significantly higher in participants with ISCED 5/6 compared with participants with ISCED 1/2 (*p* < 0.001), and was higher in participants with ISCED 5/6 compared with the ISCED 3/4 group (*p* < 0.001; 3/4 versus 1/2; *p* = 0.26). Because *g* has an SD of 1, the adjusted mean *g* values shown in Table 2 translate to a 0.63 SD higher *g* and a 0.50 SD higher *g* in ISCED 5/6 compared with ISCED 1/2 and ISCED 3/4 respectively in this model.

**Table 2:** Associations of education and occupation with the global ability factor *g*

	Model 1: adjusted for age, sex			Model 2: adjusted for age, sex, pre-morbid IQ			Model 3*: adjusted for age, sex, pre-morbid IQ, depression score		
	Mean *g* (95% CI)	*p*-value	partial eta^2^	Mean *g* (95% CI)	*p*-value	partial eta^2^	Mean *g* (95% CI)	*p*-value	partial eta^2^
Education		< 0.001	0.076		< 0.001	0.036		< 0.001	0.037
ISCED 1/2	−0.26 (− 0.44, − 0.07)			− 0.13 (− 0.32, 0.06)			− 0.09 (− 0.29, 0.10)		
ISCED 3/4	− 0.13 (− 0.24, − 0.02)			−0.10 (− 0.21, 0.02)			−0.09 (− 0.20, 0.03)		
ISCED 5/6	0.37 (0.26, 0.48)			0.28 (0.17, 0.40)			0.30 (0.18, 0.41)		
Occupation		< 0.001	0.037		0.058	0.015		0.180	0.011
Semi−/unskilled	−0.18 (−0.42, 0.07)			−0.07 (− 0.31, 0.17)			0.06 (− 0.19, 0.31)		
Skilled manual	−0.19 (− 0.36, − 0.02)			−0.08 (− 0.25, 0.09)			0.08 (− 0.26, 0.10)		
Skilled nonmanual	0.04 (−0.09, 0.16)			0.03 (−0.10, 0.15)			0.05 (−0.07, 0.18)		
Managerial	0.28 (0.04, 0.51)			0.28 (0.05, 0.51)			0.20 (−0.03, 0.44)		
Professional	0.27 (0.13, 0.41)			0.17 (0.03, 0.31)			0.18 (0.04, 0.33)		

*N* = 623. Each of the two rows shows a separate ANCOVA model for education and occupation respectively with global ability factor *g* as dependent variable

Mean *g* shows estimated marginal means (95% CI). Depression scores were log-transformed. ISCED, International Standard Classification of Education. *n = 581

Model 1: ISCED 1/2 versus 3/4, 0.13 SD difference in *g*; ISCED 1/2 versus 5/6, 0.63 SD difference in *g*; ISCED 3/4 versus ISCED 5/6, 0.50 SD difference in *g*

Model 2: ISCED 1/2 versus 3/4, 0.03 SD difference in *g*; ISCED 1/2 versus 5/6, 0.41 SD difference in *g*; ISCED 3/4 versus ISCED 5/6, 0.38 SD difference in *g*

Model 3: ISCED 1/2 versus 3/4, 0.00 SD difference in *g*; ISCED 1/2 versus 5/6, 0.39 SD difference in *g*; ISCED 3/4 versus ISCED 5/6, 0.39 SD difference in *g*

Model 1: Semi/unskilled versus skilled manual, 0.01 SD difference in *g*; versus skilled nonmanual, 0.22 SD difference in *g*; versus managerial, 0.46 SD difference in *g*; versus professional, 0.45 SD difference in *g*. Skilled manual versus skilled nonmanual, 0.22 SD difference in *g*; versus managerial, 0.47 SD difference in *g*; versus professional, 0.46 SD difference in *g*. Skilled nonmanual versus managerial, 0.24 SD difference in *g*; versus professional, 0.23 difference in *g*. Managerial versus professional, 0.01 SD difference in *g*

Model 2: Semi/unskilled versus skilled manual, 0.01 SD difference in *g*; versus skilled nonmanual, 0.10 SD difference in *g*; versus managerial, 0.35 SD difference in *g*; versus professional, 0.24 SD difference in *g*. Skilled manual versus skilled nonmanual, 0.11 SD difference in *g*; versus managerial, 0.36 SD difference in *g*; versus professional, 0.25 SD difference in *g*. Skilled nonmanual versus managerial, 0.25 SD difference in *g*; versus professional, 0.25 difference in *g*. Managerial versus professional, 0.11 SD difference in *g*

Model 3: Semi/unskilled versus skilled manual, 0.02 SD difference in *g*; versus skilled nonmanual, 0.01 SD difference in *g*; versus managerial, 0.14 SD difference in *g*; versus professional, 0.12 SD difference in *g*. Skilled manual versus skilled nonmanual, 0.03 SD difference in *g*; versus managerial, 0.12 SD difference in *g*; versus professional, 0.10 SD difference in *g*. Skilled nonmanual versus managerial, 0.15 SD difference in *g*; versus professional, 0.13 difference in *g*. Managerial versus professional, 0.02 SD difference in *g*

For pairwise comparison *p*-values, see text

The strength of the association between education and cognitive ability was reduced when the model was additionally adjusted for pre-morbid IQ, but it remained statistically significant (Model 2; Table 2). The partial variance in *g* explained by education was 7.6% in the model without pre-morbid IQ, and it was 3.6% in the model with pre-morbid IQ. Results did not substantially change when the model was further adjusted for depression (Model 3; Table 2). In this model, participants with ISCED 5/6 had significantly higher *g* compared to the ISCED 1/2 (*p* = 0.001) and the ISCED 3/4 groups (*p* < 0.001; 3/4 versus 1/2 *p* = 0.95). Independently of age, sex, pre-morbid IQ and depression, participants in the ISCED 5/6 group on average scored 0.39 SD higher on *g* compared with ISCED 3/4 and ISCED 1/2 respectively. Post-hoc addition of occupation as a covariate to Model 3 did not substantially attenuate the association of education with *g* (partial variance explained, 3.0%; *p* < 0.001; occupation was not associated with *g* in this model, *p* = 0.93). An additional post-hoc model tested for interaction effects. With pre-morbid IQ used as a binary variable (‘<median pre-morbid IQ’ versus ‘≥median pre-morbid IQ’) and adjustment for age, sex and depression there were no statistically significant interaction effects of education*pre-morbid IQ on the dependent variable *g* (*p* = 0.74).

When Model 3 was repeated for the six individual cognitive tests, we found that education was statistically significantly associated with Paired Associates Learning, Spatial Span and Trail-Making Test-B at the Bonferroni-corrected level of statistical significance (all *p* < 0.004; Supplemental Table S1). To illustrate, after accounting for age, sex, pre-morbid IQ and depression, participants with ISCED 5/6 were 15 s faster compared with ISCED 3/4 (*p* < 0.001; 1/2 versus 5/6, *p* = 0.03; 1/2 versus 3/4 *p* = 0.38; median time to complete the test was 100 s, interquartile range 79 to 130 s across the total analysis sample). When education and occupation were entered concurrently into the model, education was no longer independently associated with Trail-Making Test-B at the Bonferroni-corrected level of statistical significance (partial variance explained 0.8% *p* = 0.03). Education was not associated with Verbal Recognition Memory, Simple Reaction Time or Grooved Pegboard (all *p* > 0.004; Supplemental Table S1).

### Association of occupation with cognitive ability

In a model controlling for age and sex, occupation was statistically significantly associated with *g,* accounting for 3.7% of variance (Model 1; Table 2). Pairwise comparison showed that the professional (*p* = 0.002) and managerial group (*p* = 0.01) had higher *g* compared with the semi/unskilled group respectively. Professionals (*p* < 0.001) and skilled nonmanual workers (*p* = 0.04) had higher *g* compared with the skilled manual group respectively. Professionals (*p* = 0.02) and managerial workers (*p* = 0.002) each also had higher *g* compared with the skilled nonmanual group.

No other group differences were observed (all pairwise *p* > 0.05). When the model was additionally adjusted for pre-morbid IQ, the association between occupation and *g* was no longer statistically significant (Model 2; Table 2). Addition of depression scores further attenuated the association (Model 3; Table 2). There were no statistically significant interaction effects of occupation*pre-morbid IQ in a model with *g* as the dependent variable and adjustment for age, sex and depression scores (*p* = 0.92).

In supplemental analyses of the six individual cognitive tests, after controlling for age, sex, pre-morbid IQ and depression, occupation was statistically significantly associated only with Trail-Making Test-B (*p* = 0.002; Supplemental Table S1). In this model, professionals were on average 17 s faster compared with the skilled manual group (*p* = 0.002). Managerial workers were on average 23 s faster compared with the skilled manual group (*p* < 0.001; all other pairwise comparisons *p* > 0.004). When education and occupation were entered concurrently into the model, occupation was not independently associated with Trail-Making Test-B at the Bonferroni-corrected level of statistical significance (partial variance explained 2.0%; *p* = 0.02). Occupation was not associated with any of the five remaining cognitive tests (all *p* > 0.004; Supplemental Table S1).

## Discussion

In this study of older adults, we found associations of education and occupation with the level of global cognitive ability. These associations were independent of age and sex, but were attenuated when further adjusted for pre-morbid IQ and they remained statistically significant only for education. These data suggest that the association of education with cognitive ability in older persons cannot fully be explained by pre-morbid IQ.

The identification of factors that predispose or protect people from age-related cognitive impairment is gaining in importance. In this context, a higher level of education or occupation has frequently been implicated as associated with a reduced risk of cognitive impairment in epidemiological studies [9, 16, 18], which is in line with our observation that a higher educational and occupational background were each associated with a relatively higher global cognitive ability in older age.

Considering the modifiable nature of education and occupation, a key issue approached in epidemiological research is whether or not the reported associations may reflect causality in the education/occupation-cognition direction, or whether they result from associations with confounding factors such as pre-morbid IQ. Here, we demonstrated that the associations of education and occupation with global cognitive ability in older age were substantially attenuated when adjusted for pre-morbid IQ, and remained statistically significant only for education. Thus, while the association of occupation with cognitive ability was largely explained by pre-morbid IQ, the association of education with cognitive ability was only to some extent explained by pre-morbid IQ but was additionally independently of pre-morbid IQ related to cognitive ability in older age. This finding is important, because it suggests that a higher educational attainment could potentially provide a cognitive reserve to prevent cognitive impairment in older age.

For occupation, findings similar to our own have been reported for the Lothian Birth Cohort 1936 [48]. In that study, the effect sizes for the association of occupational complexity with cognitive ability at age 70 was reduced by 50 to 66% when IQ at age 11 was controlled for [48]. This is a similar reduction in the variance explained by occupation as observed in our study when we adjusted for our vocabulary-based estimate of pre-morbid IQ. Overall effect sizes were larger in the Lothian Birth Cohort compared with our analysis, which may be due to the fact that the study included in-depth assessment of occupational complexity whereas we categorized into relatively coarse occupational groups. In our analysis, the association of occupation with global cognitive ability was further reduced when depression was controlled for and reduced towards null following additional adjustment for education. In addition to *g,* among the six individual cognitive tests, this pattern of results was similarly observed for Trail-Making Test-B. Overall, this suggests a very limited association of occupation with late-life cognitive ability when accounting for age, sex, pre-morbid IQ, depression and education.

In contrast, pre-morbid IQ did not fully explain the association of education with cognitive ability. To some extent a higher level of education predicted later-life cognitive ability irrespectively of what people had cognitively ‘started off with’. Similar unique contributions of education to late-life cognition have previously been reported [49, 50]. Yet, the severe attenuation of effect sizes following adjustment for pre-morbid IQ, which can be indicative of some degree if reverse causality with higher-ability individuals selecting higher education [51], too, are representative of the research literature. For instance, a recent analysis of a US cohort found that associations of education with late-life cognition became less consistent and were weakened when cognitive function around age 20 was controlled for [21]. Similar observations have been reported in samples of patients with Parkinson’s disease [19] and Alzheimer’s disease [20]. This inconsistency in the field could in part stem from relatively small effect sizes linking education to late-life cognition. Here, the association of higher education with higher late-life cognitive ability was also not confounded by depression at the time of testing and was independent of occupation. These results might suggest some (albeit relatively minor) unique contribution of education to cognitive ability in older age beyond its relationships with pre-morbid IQ, depression and occupation. Further, pairwise comparison determined that the association of education with cognitive ability was driven by highly educated individuals: the group with tertiary education had higher cognitive ability compared with the other two educational groups, whereas there was virtually no difference in cognitive ability between the two groups with primary/lower-secondary and upper secondary/post-secondary non-tertiary education in the fully adjusted model.

It may be possible that a tertiary education to some extent promoted cognitive ability and may have equipped individuals with an ability to compensate for age-related neuropathological damage, for instance through upregulation of existing and/or recruitment of novel brain networks [7, 52]. A causal relationship is supported by results from Mendelian randomization studies utilizing genetic variants as indicators of exposures [53] and by quasi-experimental studies on the effects of policy change. For instance, following an increase of compulsory schooling from 7 to 9 years in Norway, the IQ increased and more so than was to be expected from the naturally occurring rise in IQ since the beginning of cognitive testing [54]. Across 11 studies of this type, a meta-analysis determined that policy change indeed influenced cognitive ability [50]. Our observation of a beneficial role particularly of tertiary education without any difference between the lower two educational groups contrasts with those observations of effects of an increase in compulsory, i.e. primary, education, but may be due to our sample characteristics. Our lower educational groups may not be representative of the general lower-education population.

We are not in a position to determine the precise mechanisms or any potential causal relationships linking education to old-age cognitive ability in our study based on the present observational data, but our findings suggest that the associations were not fully driven by reverse causality with higher-ability individuals selecting a higher education [51] We cannot rule out that our estimation of pre-morbid IQ around age 30 was also to some extent the result of educational attainment however. For this reason, studies with data on pre-morbid IQ obtained prior to the variation in schooling, i.e. before children enter individual educational paths (e.g., at age 11 [55]), are key to fully characterize the relationship of education with lifespan cognition.

Interestingly, in addition to *g*, education was associated with tests of verbal and non-verbal memory and with Trail-Making Test-B. Previous studies had similarly implicated memory as associated with education [21, 56], in some even with pre-morbid IQ controlled for [21], whereas education or cognitive reserve composite measures that include education have frequently been found not to be associated with measures of processing speed [21, 49, 56–58]. These types of observations have led to the hypothesis that a longer time spent in formal education may enhance cognitive performance in areas such as memory or reasoning in older age but has no effect on more elementary abilities such as processing speed [49]. Here, we suggest that our finding for Trail-Making Test-B may be due to its executive function component, which is a higher-order, frontal ability, rather than its processing speed component. In support of previous literature, Simple Reaction Time (a more 'pure' measure of processing speed) was indeed not associated with cognition in our analysis.

 Future studies should attempt to determine the potential causality underlying observational reports such as our own. As trial studies on the effect of a modification of educational experience are difficult from an ethical and practical perspective, further Mendelian Randomization studies will prove useful in this context. Ultimately, depending on effect sizes, which may in fact be relatively small according to our and others’ cross-sectional [21] and prospective analyses [58], recommendations could be made on the optimal educational structure for preservation of cognitive ability into older age*.* Research is also needed into any practical implications. Education is a highly individual experience dependent on preference and access, and any top-down strategies to promote education are likely complex and costly. Additionally, in ageing societies, a delay in young adults entering the workforce can be problematic and so an investigation of any differential (potentially causal) effects of earlier versus later education during the lifespan on late-life cognition could be useful. Here, we used baseline data from a sample of older adults scheduled for surgery within the next few days. Whether or not a higher education and occupation protected our patients from developing post-operative cognitive dysfunction, which at least for education previous studies appear to suggest [59], remains to be seen.

Strengths of our study include use of a large sample size and a comprehensive cognitive test battery that covered several age-sensitive cognitive domains in addition to a composite measure of global ability. Yet some limitations need to be considered. We used a cross-sectional design and with no access to actual pre-morbid IQ obtained prior to a variation in schooling (e.g., [55]) resorted to a vocabulary-based estimate, though previous analyses of birth cohorts comparing such estimates with measured pre-morbid IQ have validated the approach [2]. Selection bias preferring individuals with a higher cognitive ability, education and occupation was indicated by a relatively high mean pre-morbid IQ as well as by a disproportionally high number of participants in the higher educational and occupational groups. This is a problem shared by many studies on cognitive ageing [60]. Our results may therefore not translate to the general older population. We recruited participants from a large age range subject to cohort effects on cognitive ability [61]. Between-test differences in terms of measurement scales will also have affected the comparison of education and occupation associations across cognitive tests. For instance, scores on Spatial Span were relatively coarse (range 0 to 9) whereas for the timed tests, measures were presented in more fine-grained scales of seconds or milliseconds. Further, participants were asked about their current occupation or their most recent occupation before retirement, which may not represent their highest level of occupational attainment. Some individuals may achieve a high level of occupation but change occupational category later in late (e.g., moving from skilled manual to skilled non-manual; or from managerial to skilled non-manual). All of these factors may have introduced noise to our analysis of occupation and may have weakened its relationship with cognitive scores. Finally, we assessed the independence of education and occupation associations with cognitive ability from pre-morbid IQ through statistical adjustment.Because pre-morbid IQ itself was associated with education and occupation, its addition to these models will have led to a lower proportion of variance being free to be accounted for by these independent variables [62], which may have contributed to the observed reduction in effect sizes observed in this modeling step. Concurrent addition of education and occupation into a single model in our post-hoc analyses, too, was limited by their correlation.

## Conclusion

Here, we have contributed to an ongoing research field that has yet to fully elucidate the association of cognitive reserve with cognitive ability in older age. An association of current or (if retired) previous occupation with the level of cognitive ability in older age was largely explained by pre-morbid IQ. In contrast, tertiary education had some, albeit relatively minor unique contribution to a higher cognitive ability in older age that was independent of its relationship with pre-morbid IQ, depression as well as occupation. The difference in cognitive ability between the two lower educational groups was relatively flat. Further research is needed to determine whether causality underlay out observations, which would imply a potential benefit of increasing education on reducing cognitive risk in older age. 

## Supplementary Information

**Additional file 1: Table S1**. Associations of education and occupation with scores on individual cognitive tests

## Data Availability

The datasets generated and/or analyzed during the current study are not publicly available due to threats to participant privacy.

## Abbreviations

BioCog: Biomarker Development for Postoperative Cognitive Impairment in the Elderly study
DART: Dutch Adult Reading Test
*g*: Global ability factor
GDS: Geriatric Depression Scale
ISCED: International Standard Classification of Education
MMSE: Mini Mental State Examination
MWT-A: Mehrfachwortschatztest A
PCA: Principal component analysis
