# Supplementary material for: Contribution of IQ in young adulthood to the associations of education and occupation with cognitive ability in older age
Source: BMC Geriatr. 2021 Jun 5;21:346. doi: 10.1186/s12877-021-02290-y (PMC8180039; doi:10.1186/s12877-021-02290-y)
Supplement: Supplementary file 1 — Additional file 1: Table S1. Associations of education and occupation with scores on individual cognitive tests [file 12877_2021_2290_MOESM1_ESM.docx]

Supplemental Table S1: Associations of education and occupation with scores on individual cognitive tests

|  | Model 3: adjusted for age, sex, pre-morbid IQ, depression score | | | | | | | | | | | | | | | | | |
| --- | --- | --- | --- | --- | --- | --- | --- | --- | --- | --- | --- | --- | --- | --- | --- | --- | --- | --- |
|  | Paired Associates Learning  Possible score range 0 to 26 | | | Verbal Recognition – free recall  Possible score range 0 to 12 | | | Simple Reaction Time* (milliseconds) | | | Spatial Span  Possible score range 0 to 9 | | | Trail-Making Test-B* (seconds)  Maximum 300 seconds | | | Grooved Pegboard* (seconds)  Maximum 300 seconds | | |
|  | Mean score (95% CI) | p-value | partial eta^2^ | Mean score (95% CI) | p-value | partial eta^2^ | Mean score (95% CI) | p-value | partial eta^2^ | Mean score (95% CI) | p-value | partial eta^2^ | Mean score (95% CI) | p-value | partial eta^2^ | Mean score (95% CI) | p-value | partial eta^2^ |
| Education  ISCED 1/2  ISCED 3/4  ISCED 5/6 | 12.5  (11.6, 13.5)  13.3  (12.8, 14.0)  14.6  (14.0, 15.2) | **0.001** | **0.023** | 5.8  (5.4, 6.2)  5.6  (5.4, 6.4)  6.1  (5.9, 6.4) | 0.009 | 0.016 | 323  (304, 342)  310  (299, 321)  308  (298, 319) | 0.415 | 0.003 | 4.6  (4.1, 4.9)  4.7  (4.6, 4.8)  5.0  (4.9, 5.2) | **0.001** | **0.024** | 109  (98 116)  111  (106 116)  96  (91 100) | **<0.001**** | **0.030** | 91  (85, 97)  94  (90, 98)  91  (87, 95) | 0.475 | 0.003 |
| Occupation  Semi-/unskilled  Skilled manual  Skilled non-manual  Managerial  Professional | 13.8  (12.6, 15.1)  12.8  (11.9, 13.7)  13.8  (13.2, 14.4)  14.2  (12.9, 15.2)  14.3  (13.6, 15.1) | 0.134 | 0.012 | 5.9  (5.5, 6.4)  5.5  (5.1, 5.8)  5.9  (5.6, 6.1)  5.8  (5.3, 6.2)  6.1  (5.9, 6.4) | 0.067 | 0.015 | 306  (285, 330)  304  (288, 321)  313  (301, 325)  313  (292, 335)  314  (301, 329) | 0.872 | 0.002 | 4.9 (4.6, 5.2)  4.8 (4.5, 5.0)  4.8 (4.6, 4.9)  4.9 (4.6, 5.1)  4.9 (4.7, 5.1) | 0.807 | 0.003 | 109  (99, 121)  116  (108 125)  102  (97, 108)  93  (85, 102)  99  (94, 106) | **0.002**** | **0.029** | 94 (86, 102)  86 (81, 92)  95 (92, 100)  89 (82, 97)  93 (88, 98) | 0.131 | 0.012 |

N=581. Each of the two rows and six columns show separate ANCOVA models associating education and occupation respectively with the individual cognitive tests. Means show estimated marginal means (95% CI). Depression scores, Simple Reaction Time, Trail-Making Test-B and Grooved Pegboard were log-transformed. ISCED, International Standard Classification of Education. *means shown are geometric means; higher values mean worse performance on these tests. **when education and occupation were entered concurrently into the model, their associations with TMT-B were no longer statistically significant at the Bonferroni-corrected level p<0.004 (education, partial eta^2^ 0.008, p=0.03; occupation, partial eta^2^ 0.020, p=0.02). Bold font, statistically significant following Bonferroni correction (p<0.004).
